# The Impact of Individual Behaviors and Governmental Guidance Measures on Pandemic-Triggered Public Sentiment Based on System Dynamics and Cross-Validation

**DOI:** 10.3390/ijerph18084245

**Published:** 2021-04-16

**Authors:** Hainan Huang, Weifan Chen, Tian Xie, Yaoyao Wei, Ziqing Feng, Weijiong Wu

**Affiliations:** 1 School of Economics, Management and Law at the University of South China, Hengyang 421001, China; hhn0113@outlook.com (H.H.); 2014002159@usc.edu.cn (Y.W.); fzq@stu.usc.edu.cn (Z.F.); 2 Information Sciences and Technology at The Pennsylvania State University, State College, PA 16802, USA; weifan@psu.edu; 3 School of Management, Guangdong University of Technology, Guangzhou 510520, China; wuweijiong@gdut.edu.cn

**Keywords:** pandemic, public sentiment, system dynamics, cross-validation, simulation and control

## Abstract

Negative online public sentiment generated by government mishandling of pandemics and other disasters can easily trigger widespread panic and distrust, causing great harm. It is important to understand the law of public sentiment dissemination and use it in a timely and appropriate way. Using the big data of online public sentiment during the COVID-19 period, this paper analyzes and establishes a cross-validation based public sentiment system dynamics model which can simulate the evolution processes of public sentiment under the effects of individual behaviors and governmental guidance measures. A concrete case of a violation of relevant regulations during COVID-19 epidemic that sparked public sentiment in China is introduced as a study sample to test the effectiveness of the proposed method. By running the model, the results show that an increase in government responsiveness contributes to the spread of positive social sentiment but also promotes negative sentiment. Positive individual behavior suppresses negative emotions while promoting the spread of positive emotions. Changes in the disaster context (epidemic) have an impact on the spread of sentiment, but the effect is mediocre.

## 1. Introduction

In January 2020, an outbreak of COVID-19 began in Wuhan, China; this virus eventually spread rapidly to more than 200 countries. Since then, there have been over 79.2 million cases and 1.7 million deaths reported [1]. Closing educational institutions and face-to-face businesses, limiting gatherings to 10 people or less, and strict stay-at-home orders are many non-pharmaceutical interventions (NPIs) that governments put in place in an attempt to control the COVID-19 pandemic. However, NPIs can also indirectly create new problems: negative public sentiment and misinformation.

During the period of home quarantine, physical interpersonal communication is blocked and social networks become an essential communication channel. Due to the single source of information and fear of unknown viruses, a large number of negative online public sentiment incidents and misinformation spreading broke out during the pandemic, such as the Shuang-huang-lian panic buying incident in China, the 5G-caused spread of coronavirus in the UK, and more. Past empirical research results have shown that public health emergencies can trigger more negative public sentiment and misinformation, generating negative emotions and affecting psychological and physical health. Negative emotions may damage the immune system, leading to long-term infections and delayed wound healing [2]. During an epidemic, if the government fails to guide public xenophobia, it may lead to public blame of the government [3] (e.g., black Africans blaming AIDS(Acquired Immune Deficiency Syndrome) on the white governments of non-African countries [4,5]), and people who do not trust the government may not participate in beneficial public health programs (e.g., government-mandated vaccination programs [6,7,8]). Based on the above research conclusions, how to guide the negative public sentiment in time and improve the individual’s emergency psychology preparedness is the central topic of the research.

The study of negative public sentiment and misinformation usually uses an empirical and model-based approach. Public emotions and cognition are usually measured by retrospective questionnaires, such as the Oxford Happiness Inventory [9], Symptom Checklist 90 [10] and Likert Type Attitude Scale [11]. Using scales, questionnaires, and second-hand data to build statistical models is an important method for analyzing public psychology. On the one hand, a large number of scholars have studied the influence of public sentiment on the external factors. For example, Gilles et al. found that public trust in medical organizations was related to vaccination behavior and predicted the public’s H1N1(2009 swine flu pandemic) vaccination behavior in 2009 [7]. Bogart et al. found a strong relationship between AIDS conspiracy and medical non-adherence among African Americans [6]. Hong et al. revealed the relationship between public trust in the government and individual public health emergency preparedness [12]. On the other hand, many scholars have studied the intrinsic generation and evolutionary logic of public emotions. For example, Li et al. used social platform data to study the evolution of public psychology before and after the declaration of the COVID-19 epidemic [13]. Apuke et al. analyzed the internal motivation of sharing fake news from psychological factors such as “altruism”, “entertainment”, and “socialization”, based on the Uses and Gratification framework [14]. Hong et al. studied the relationship between political news in different forms of media and public happiness psychology [15]. Differing from empirical research, model-based research focuses on public psychology prediction, policy evaluation, communication mechanism, complex system behavior, etc., and have unique advantages in considering complex, nonlinear, and self-loop. For example, Liu et al. established a contagion diffusion model for public opinion simulation based on game theory to reveal the contagion path of public opinion [16]. Naskar et al. studied the public sentiment propagation characteristics of Twitter users based on the Russell model and TESC technology [17]. Xie et al. proposed a parallel evolution and response decision-making framework of public emotion based on system dynamics and parallel control management theories, which is a real-time decision-making method to simulate and control public sentiment [18].

**Figure 1:**
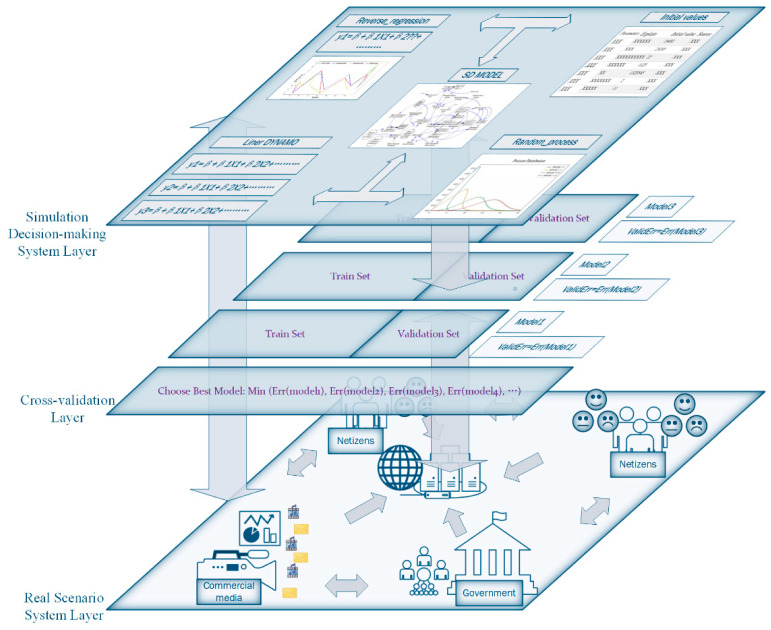
Cross-validation modeling framework for public sentiment system.

A review of literature in recent years reveals that many fields, including public health [13], business management [19], medical management [7], communication media [17], emergency management [18], and economics [20] have conducted research on public psychology, but there is still a certain lack in model construction, validation, and applicable measures analysis. Considering that a single linear model is not sufficient to reflect the real social complex system of nonlinear multiple information and self-feedback, a system dynamics method is introduced on the basis of the linear model, so as to consider the nonlinear characteristics and avoid the subjectivity of the parameter. In addition, considering the lack of data and the model validation, we introduce the cross-validation method to improve the effectiveness of the model. Finally, considering the stochastic characteristics of the real world, this article introduces a random process on the basis of the model to make the model more suitable for real situations. Figure 1 shows the research idea map of this article.

## 2. Materials and Methods

### 2.1. Cross-Validation Modeling Framework for Public Sentiment Based on System Dynamics

Cross-validation is a model selection method that can be used to directly estimate Generalization Error. This method can be used for model verification and model effectiveness improving. Because of its simplicity, it is widely used in the machine learning field [21,22]. Usually, the internal relationships of a public sentiment system, which describe the operating rules and determine the validity of simulation results, are difficult to verify. Therefore, the “Cross-validation modeling framework for public sentiment based on system dynamics” (CVMFPS) is proposed as a guideline to solve this problem (Figure 1). According to the “scenario-response”-based emergency management paradigm [23], and combined with the cross-validation method, this model consists of three parts: the real scenario system layer, the cross-validation layer, and the simulation decision-making layer.

#### 2.1.1. Real Scenario System Layer

As the source of information, real scenarios are the basis for decision-making as well as the targets of public sentiment control. The original events, sentiment disseminators and sentiment regulators are essential elements of the real scenario system. The original events (such as public health emergencies, government scandals and mistakes) may easily trigger relevant public sentiment. Public sentiment disseminators include the media, netizens, and others. The media triggers and influences the processes of public sentiment propagation through reporting and directing the news. In addition, netizens use social networks to express and communicate their own opinions which results in the continued diffusion and evolution of public sentiments. Because collective behaviors of the netizens comprehensively effect their attitudes towards source events, their support or opposition are essential factors for the government in making an efficient response decision. Generally, the government response departments dealing with the emergencies or mistakes assume the greatest responsibilities as public sentiment regulators. By taking measures such as holding lectures and seminars, press conferences on the events, and by releasing positive news, they may supervise, guide, and even control the development of the public sentiments.

#### 2.1.2. Cross-Validation Layer

Using data for training model without testing, even if training error is small, does not mean that the model is correct. The model fits well on the training set, but the actual predictions are poor due to overfitting problems. In order to overcome this problem, the cross-validation method was proposed. The idea of this method is to divide the complete data set D (Equation (1)) into two parts randomly according to a certain proportion. The data set used to train the model is called the training set D^t^ (Equation (3)), and the data set used to test the model is called the validation set D^v^ (Equation (2)). In the Equations, s(1) represents the first data that output randomly, m represents the data size of the validation set, and *n* represents the total number of data sets. Since the training data and the validation data are not the same, the generalization error of the model is estimated on new data, and it is closer to the real generalization error. In the System Dynamics model, the dynamo equation reflecting the specific influence relationship between variables is constructed in a mathematical way, but due to the complexity, randomness and instability of the social system, the dynamo equation cannot be like accurately calculated like a physical model, so the quantitative relationship and directional relationship between the variables reflected by the dynamo equation under the social system need to be verified. Therefore, through the combination of variables, different internal model structures and mathematical equations are constructed, the cross-validation method is used to calculate the error of these models on the verification set and select the best model that is closest to the real situation, and this is an effective way to build models when the data is insufficient:

D = {D_1_, D_2_, D_3_, D_4_…D_n_}, D = D^t^ + D^v^ (1)

D^v^ = {D_s(1)_, D_s(2)_, D_s(3)_, D_s(4)_ … D_s(m)_} (2)

D^t^ = {D_s(m+1)_, D_s(m+2)_, D_s(m+3)_, D_s(m+4)_ … D_s(n)_} (3)

#### 2.1.3. Simulation Decision-Making System Layer

From the cross-validation layer, we have well-structured system dynamics internal structures and dynamo equations. The dynamo equation is used to represent the specific relationship between various variables in the SD model. By adding stochastic process to dynamo equations, the SD model can evolve autonomously based on the random results at each time, its simulation results will be closer to reality, and the use of stochastic process can also test the robustness of the SD model. For example, the Poisson distribution can represent the frequency of occurrences of random events in a unit time and plug in each occurrence node, the mean frequency of occurrences of events in a unit time can be the parameter for Poisson distribution. In addition, this layer proposes a method to improve the simulation effect of the model, called Reverse Regression, which is different from linear regression (Equation (4)), the variable (*sharefactor*) in reverse regression (Equation (5)) does not have real data, this variable needs to be calculated by other independent variables and dependent variable. Reverse regression requires the *sharedfactor* to be constant during a certain event but change in different events, and also requires other variables (*othervar_i_*) and *sharefactor* can explain the main variance of y. Therefore, by iterating the *sharedfactor* data, the trend of the *sharefactor* between different subjects tends to be consistent, and the value of the *sharedfactor* can be calculated. The specific calculation process is given in Section 3.2. Finally, by inputting the initial parameters of the new public sentiment event from the real scenario system and using these methods, the final SD model can be established. Through simulation, different response strategies or policies can be tested, verified, and optimized in the simulated environment

*y* = β_0_ + β_1_ ∗ *independent* + β_i_ ∗ *othervar*_i_ (4)

*y* = β_0_ + β_1_ ∗ *sharedfactor* + β_i_ ∗ *othervar*_i_ (5)

### 2.2. Methodology

**Figure 2:**
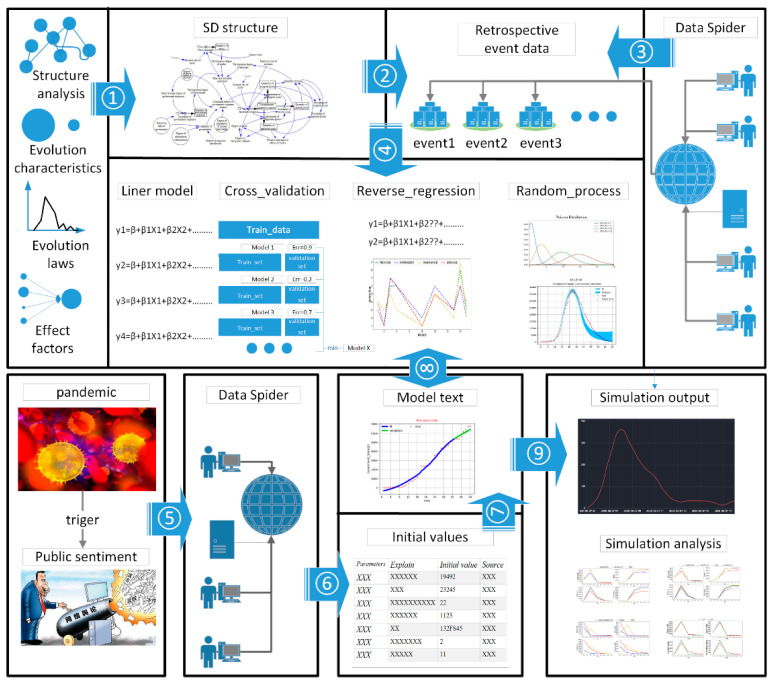
Research roadmap.

Roadmaps are helpful for decision makers to know how to use a modelling and simulation method for dealing with practical problems [24]. To implement the CVMFPS method, we developed a roadmap that describes the steps shown in Figure 2. The order in the roadmap is only for reference, and we need to use the appropriate modeling order in the face of different real-world problems. In summary, the roadmap contains a series of steps, from data acquisition, modeling to simulation and analysis.

#### 2.2.1. Decision Problem, Materials and Hypotheses

As a qualitative and quantitative decision-making method, the CVMFPS framework is applicable for response to the public sentiment without enough historical data. Therefore, the decision makers must determine what type of decision problem it is: Is there enough historical data for building a model for this event? If decision makers have enough investigable historical data, it is better to use the data-dependent statistical methods, such as Machine learning. If not, the SD (System Dynamics) simulation model with CVMFPS is a good idea. In addition, the source and measurement of data are also important issues to be considered before modeling, and for public sentiment, questionnaire data and secondary data are the main sources. There is a time lag for obtaining questionnaire data, which is often suitable for retrospective studies. Secondary data, especially the huge amount of data from social platforms, is easier to obtain and has great information potential, which is suitable for emergency research, but secondary data often has difficulty in data validation, and the common solution is to compare data from different data sources. In conclusion, it is particularly important to consider the type of data according to the model. For public sentiment, the use of web spider to obtain the latest data in real time on social networks is beneficial for SD model building and immediate policy analysis. Finally, the implementation of CVMFPS requires some prerequisite assumptions, and the fulfillment of which is a prerequisite for using the model. For public sentiment, CVMFPS often makes requirements in terms of the dissemination mechanism and simulation of sentiment.

#### 2.2.2. SD Modelling

The SD emphasizes how causal relationships among system structures can influence the behaviors and evolution processes of a system. Analysis of the boundary and structure of a public sentiment system is the first step to building the SD model. System boundaries include the basic elements of the system. The function modules of the system consist of the elements that have direct causal relationships with each other.

System boundaries, influencing factors and causal loops are important for SD. Clarifying the system boundary of the problem facilitates us to focus on the subjects of the system without getting caught in the endless circulation of causal structures of complex social systems. In addition, the system boundary specifies the scope of application. The scope of application of the model is very important for practical applications; only when the important conditions are satisfied, the simulation results of the model have practical significance, and the focus of the model is consistent with the actual problem, is it possible to propose a solution strategy. The public sentiment system can be divided into original events and three interactional modules according to the different roles of the sentiment disseminators: the media module, the government module, and the netizen module. Therefore, the boundary of the public sentiment model should be within netizens, commercial media, and government. The purpose of the internal influence factor analysis of the system is to find out the relationship between each element. By distinguishing the independent and dependent variables, we can find a series of causal chains, and by transforming the dependent and independent variables, the influence is transmitted downward. When the lower end of the causal chain is connected to the upper end, the causal loop is thus generated [25]. The causal loop is the key to the autonomous evolution of the SD model, and the system has the ability to generate data autonomously when the effects of variables are fed back through different variables [26]. Usually, due to the wide distribution of Netizens and the profit-seeking nature of commercial media seeking exposure, these two types of subjects are the first to capture the events. The commercial media follows up on the events, the netizens express their opinions and generate emotions about them, and then, the stakeholders of the events (usually the government), depending on the nature of the events and the attitude of the public, responds accordingly. Moreover, the public, the commercial media, and the government will behave according to the behavior of other subjects, so interaction between the three types of subjects will form multiple causal loops that will eventually dominate the development of public sentiment.

Causal loop diagrams aid in visualizing a system’s structure and behavior and in analyzing the system qualitatively. By analyzing the variables in the causal loop, we can construct more specific influence relationships, which will then involve specific mathematical formulas. To perform a more detailed quantitative analysis, a causal loop diagram is transformed into a stock and flow diagram. A stock is the term for any entity that accumulates or depletes over time, using an ordinary differential equation. A flow is the rate of change in a stock. In addition, in the stock flow paradigm, there are Auxiliary variables, relational linkages, etc. The judicious use of these tools will reduce the complexity of modeling. Moreover, it is also necessary to estimate the initial parameters. Usually, the parameters and initial conditions of the equations can be estimated using statistical methods, expert opinion, market research data, or other relevant sources of information [27]. Finally, converting the system stock flow paradigm into level, rate and auxiliary equations is the key step to run the model. In addition to constructing specific model structures and equations, we also nest random functions on equations. On the social network platform, the number of posts or reposts of netizens and media per unit time obeys a poisson distribution with λ as the mean value of posts. Therefore, for each time period, the number of posts of netizens or media is nested in a Poisson distribution (Equation (6)):

*Posts = poisson (λ = mean(posts))* (6)

To construct specific mathematical equations, it is necessary to choose an appropriate expression method for the variable relationships. In complex social systems, the relationships between social variables cannot be constructed as precise equations can be constructed for engineering systems. Faced with the randomness, complexity, and incomplete predictability of social systems, it is a common and extremely practical method to estimate the relationships between variables in statistical models. There are quite a few statistical models that can reflect the relationship between variables, such as lin ear regression (LR), logistics regression, SVM (Support Vector Machine), neurl network, LSTM (Long Short-Term Memory), etc. The LR model is widely used by social science fields, such as economics and management. It is simple to operate and can predict continuous values. Although it is a linear model, the introduction of system dynamics can alleviate the linearity problem, so the LR model is used in this paper. Another purpose of using LR is to enable the reverse regression method. *Sharefactor* indicates that different variables are collectively influenced by *sharefactor*, and that *sharefactor* does not change within the same event (a short period of time) but change within different events (a long period of time). In the public sentiment system, the nature of the event itself (degree of harm to society, realistic fashion trends, etc.) and the nature of the netizens (education, income, family, etc.) will jointly influence the number of postings by netizens. The pseudo-code for the implementation process is detailed in Appendix A (Algorithm A1). The NRI in pseudocode 1 denotes the minimum times that the values in *sharefactor* are randomly varied so that the *othervar* and *sharefactor* variables can explain the main variance of the dependent variable. NI indicates how many sets of *sharefactor* are obtained; the larger the NI, the more likely it is to find the correct *sharefactor*, but there will be a large number of similar *sharefactors*. Pseudocode 1 finally outputs the most similar *sharefactor* between different dependent variables, and by drawing graphs of the *sharefactor* and observing the mutual trend, we can determine whether the *sharefactor* can be used or not. To obtain the value of *sharefactor* using linear regression, the following conditions need to be satisfied:

(1) The *sharefactor* values derived from different variables need to be verified against each other, and only if the trends are consistent can they be adopted;
(2) *Othervar* variables need to contain the main factors that can influence the dependent variable except *sharefactor*, i.e., *othervar* and *sharefactor* variables can explain the main variance of the dependent variable;
(3) Select the time period with less interference from external factors for reverse regression method, which facilitates the correct finding of *sharefactor* values;
(4) Assuming the value range of the *sharefactor* in advance, it is generally 0 to 10, −1 to 1 or 0 to 100, depending on requirements

#### 2.2.3. Cross-Validation

When studying social problems, there are complex interactions within SD models, which make it impossible to construct accurate mathematical equations. When analyzing the independent variables of a dependent variable, we can’t determine whether certain variables are independent or not. Reviewing the previous research results will identify some variables, but usually those are incomplete. The SD models constructed with incomplete variables will amplify the bias through feedback loops, which leads to unreliable simulation results. Therefore, we use the cross-validation method to select these uncertain variables and to verify the generalization ability of the mathematical equations. The pseudo-code for the implementation process is detailed in Appendix A (Algorithm A2). To run Algorithm A2, it is necessary to give the deterministic and uncertain variables in advance. One or more uncertain variables are selected at a time for regression on the basis of the deterministic variables. Then, the training set is used for training and the validation set is used to verify the training results. By adding different combinations of uncertain variables each time, we can get many models and select the model with the smallest validation set error as the mathematical model of SD. The data in the training and validation sets are in events as units, and each event is in units of time, so that when the data is split, the complete event, rather than the unit time of the events, can be used as the validation object. This allows for a better validation effect of the generalization ability of the model. Finally, the use of the cross-validation method also needs to satisfy the premise that the error of the model trained from the training set is small.

#### 2.2.4. Simulating and Decision Analysis

Some advanced SD software tools, such as Vensim (Ventana Systems Inc., Harvard, MA, USA), STELLA (Isee Systems, Lebanon, PA, USA) and Anylogic (The AnyLogic Company, Oakbrook Terrace, IL, USA), are able to help decision-makers construct, run and analyze the SD simulation models of public sentiment systems to create optimized response policies and solutions in a graphic and visual way [28]. However, these software packages can have limited functions; if you want to apply new algorithms or use unique equations, they will need to be implemented using your own programming. To propose suitable response solutions, the relevant decision analysis process should include two aspects [18]. First, response strategies setting. In constructing the SD model, control variables need to be considered in advance, and for public sentiment, we can set control variables from three perspectives: public, government, and commercial media. The public side can be started from the personal side, such as education, science popularization, the degree of trust in the government, etc. The government side is variables such as response time, information transparency, science popularization, etc. Commercial media would be variables such as speed of reporting, dissemination, etc. In addition, we also need response strategy testing. The effects of different strategies can then be applied by reviewing the simulation results. By adjusting control variables, we can achieve the expected results.

### 2.3. Empirical Research

Roadmaps are helpful for decision makers to know how to use a modelling and simulation method for dealing with practical problems [24]. To implement the CVMFPS method, we developed a roadmap that describes the steps shown in Figure 2. The order in the roadmap is only for reference, and we need to use the appropriate modeling order in the face of different real-world problems. In summary, the roadmap contains a series of steps, from data acquisition, modeling to simulation and analysis.

#### 2.3.1. Data Source

We used the top public sentiment events on Sina Weibo about COVID-19 from 25 January to 20 April 2020 in mainland China as samples [29]. The Sina Weibo contained more than 1.16 million active Weibo users. Weibo is a popular platform to share and discuss individual information and life activities, as well as celebrity news in China [30]. In this paper, we use third-party python libraries such as selenium, bs4 and urllib to write crawler programs to collect relevant data from government media, commercial media, and netizens, including the number of posts, blog ID(Identity document), the number of followers, posting content, the number of likes, number of comments, posting time, etc. A total of 15 online public sentiment events were collected during the period and used as historical cross-sectional data for equation construction within the SD model, the data description is shown by Table 1. In addition, we use the new event “Picked up the son from Wuhan to Jingzhou during the city closure”, a local government official’s epidemic prevention failure that occurred on February 14, as the simulation object of the SD model to test the feasibility and validity of the model. The data collected in this paper were cross-checked by Tencent WeChat subscriptions platform (Tencent, Shenzhen, China) [31] and the third-party ZhiWeiData platform (ZhiWeiData, Beijing, China) [32], and the results of the three-party data were consistent.

#### 2.3.2. SD Modelling

The COVID-19 pandemic that broke out in early 2020 shattered the public’s sense of normalcy. In the early stages of the outbreak, people used the Internet to keep an eye on the dynamics of the outbreak in the face of the rapidly spreading virus. During this period, several public sentiment incidents erupted on the internet, most of them as a response to government negligence or individual citizens not following orders. For example, a traveler who returned from Thailand did not comply with the epidemic prevention guidelines, Wuhan government officials failed to effectively ensure normal life for residents during home quarantine, etc. Taking these events as cases, we can analyze the boundary, structure and evolution mechanism of the public sentiment system and build a relevant qualitative causal loop diagram model. This model is divided into three main modules and two scenarios: the commercial media module, the netizen module, the government module, pre-response scenario, and post-response scenario.

System boundary and prerequisite assumptions. The interactions among three subjects—netizens, commercial media and government constitute the boundary of the public sentiment system, and factors outside of these subjects are not studied in this paper. In addition, this model requires the following prerequisites to be met:

(1) Public sentiment events are the first to erupt on the Internet.
(2) Different public sentiment events are independent of each other.
(3) When a negative event is revealed and not properly handled, the public will develop negative sentiment.
(4) Positive public sentiment will arise after the government actively and properly handles negative events.

**Figure 3:**
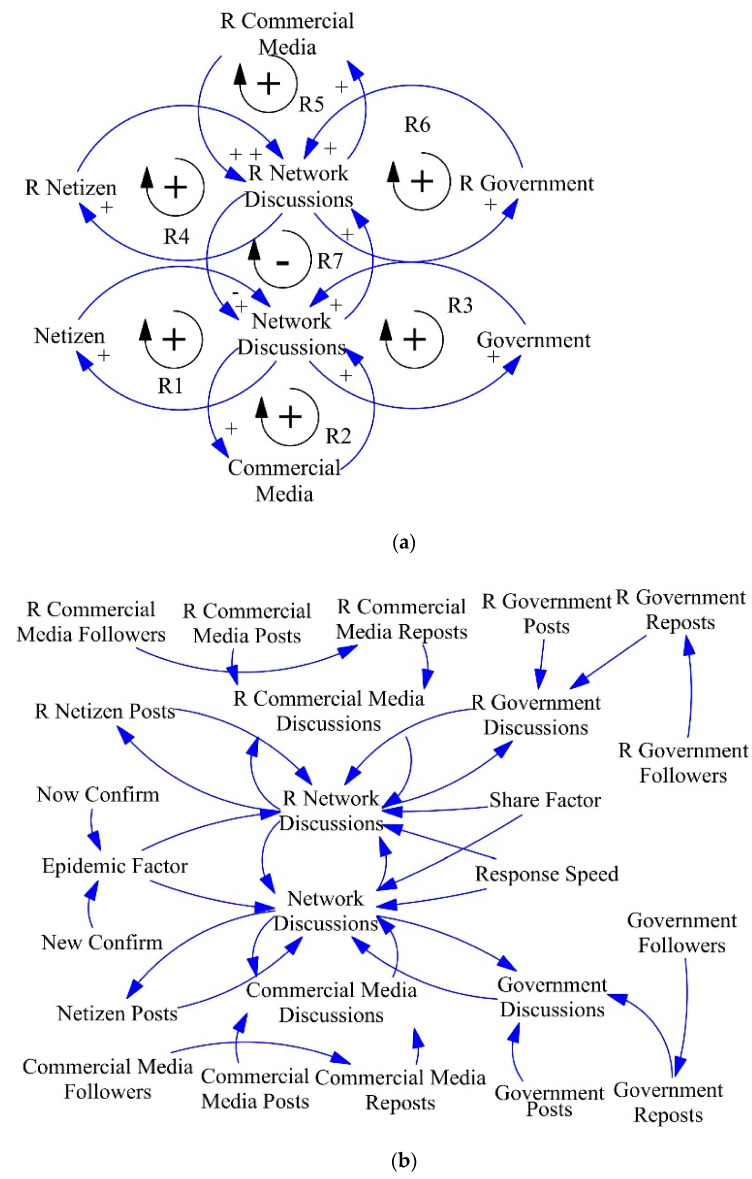
(**a**) Causal loop diagram of the public sentiment system; (**b**) Public sentiment transmission mechanism of social platforms.

The causal loop diagram of the SD model consists of three modules and two scenarios. The three modules include netizen, commercial media, and government. Netizens, commercial media, and government together constitute the total discussions online, while the herding and hotspot effects that exist in the spread of public sentiment cause discussions online to in turn promote the level of discussion among the three, thus forming multiple causal loops, as shown in Figure 3a. Netizens (commercial media and government) discussions and discussions online form a positive feedback loop. In addition, the two scenarios include pre-response and post-response scenarios. The government’s response to the incident is a turning point in the development of public sentiment. After the government’s response, netizens and commercial media turn their attention to the discussion of the content of the government’s response. The government guides public sentiment by making the right measures and spreading positive information, so that post-response public sentiment communication also forms the same mechanism as the pre-response, and the causal loops are also positive feedback loop. Finally, post-response communication of public sentiment has an impact on pre-response communication and form a negative feedback loop.

**Figure 4:**
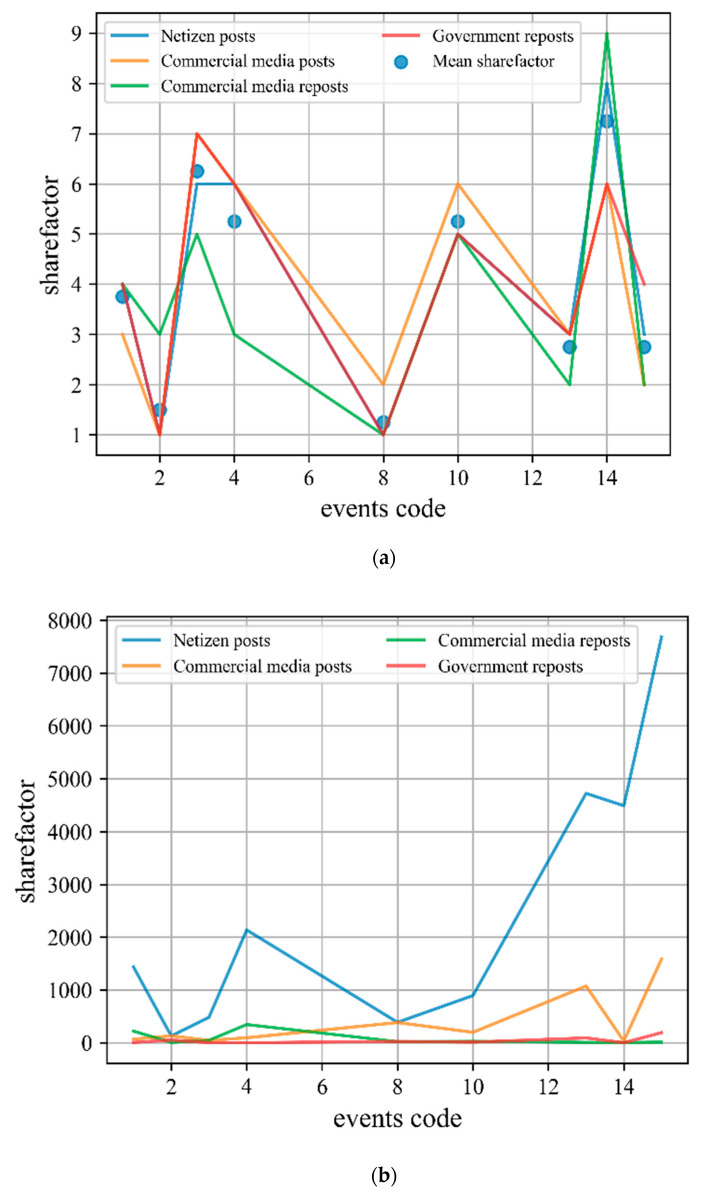
(**a**) Comparison of *sharefactor* trends between different subjects; (**b**) Comparison of raw data between different subjects.

Causal loop detailing and reverse regression. On the basis of the causal loop, we perform a causal analysis to each factor: looking for the constituents and independent variables. If necessary, further causal analysis of these independent variables and constituents can be performed. As a result, the previous causal loop is expanded into a more detailed loop diagram. In the process of detailing, it is necessary to determine the independent and dependent variables for each factor, and also to confirm whether the independent variables meet the *sharefactor* characteristics, the detailed causal loop diagram is shown in Figure 3b. Through the data, we found that most of the posts posted by netizens will not be reposted by others; in order to reduce complexity, we only consider their original posts. Commercial media includes original posts and reposts; the reposts are influenced by the number of followers of the blogger. Government media is the same as commercial media. In addition, we add Response Speed, Epidemic Factor and Share Factor in the loop. The Epidemic Factor takes into account the environmental disaster context, the Share Factor needs to be calculated by Reverse Regression, and the specific interpretation of the variables is given by Appendix A (Table A1). Compared to the middle and late period (There is a large number of non-linear relationships in middle and late periods) of an event development, in the early period public sentiment propagation mechanisms is much simpler. Therefore, we choose the first day of the event as the cross-section sample to run reverse regression, and choose netizen post, commercial media post, commercial media reports and government reposts as the cross-test subjects. Due to the fact that the first day data of some public sentiment events was missing, we selected nine events with intact data as samples. The reverse regression results are shown in Table 2 and Figure 4a. By observing Figure 4a, we can find highly similar trends among different subjects. Comparing the real data of these subjects (Figure 4b) shows that there is a high probability of finding the real *sharefactor*. Mean err in Table 2 is obtained by calculating the root mean square error of the two-by-two combination of the four subjects (Equation (7)). Where *n* denotes the number of samples and N denotes the number of the two-by-two combination of the four subjects. By averaging the *sharefactor* of these four subjects, the averaged *sharefactor* (Mean *sharefactor*) is used as the final result. The Mean *sharefactor* is then used to do a regression on a subject, and the resulting regression parameters can simplify the *sharefactor* calculation process for the new event (i.e., we only need the *othervar* and the dependent variables to calculate the *sharefactor* by these parameters):

(7) $$\mathrm{CRMS}=\frac{\sum_{j=1}^{N}\sqrt{\frac{\sum_{j=1}^{N}{\left(y_{j1}-{\hat{y}}_{j2}\right)}^{2}}{n}}}{N}$$

#### 2.3.3. Cross-Validation

**Figure 5:**
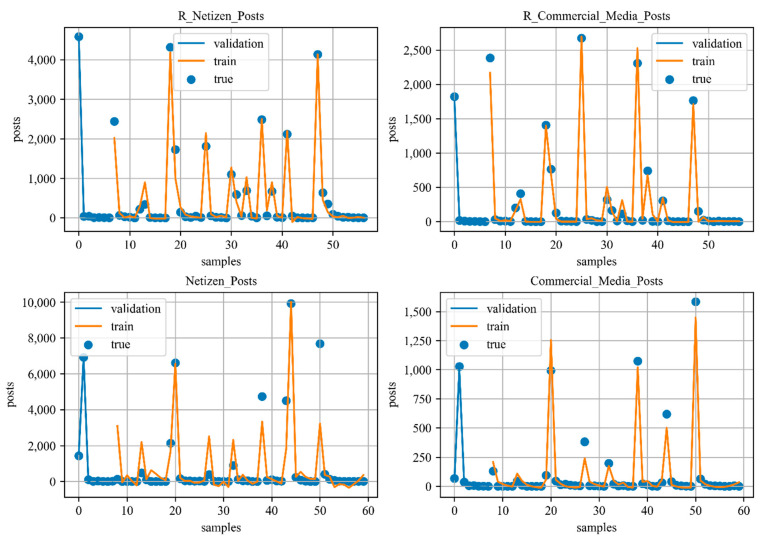
Partial training results and validation results.

After the variable analysis and data collection, the cross-validation method is applied to a total of 12 variables, and the specific process and results are shown in Table 3 and Figure 5. In general, variables that cannot be constructed by precise mathematical formulas (e.g., complex social factors) and cannot be controlled (e.g., the number of posts people make) need to be identified as dependent variables. The determination of independent variables requires an analysis of the impact relationship. At the same time, attention needs to be paid to the issue of the time sequence of occurrence, and variables that arise simultaneously or in the future cannot be included in independents. In addition, due to the error amplification effect of the causal feedback loop in the system, it is necessary to select the regression results with good training and validation GOF (Goodness of Fit) from the combination of independent variables, and the GOF of validation set can avoid the overfitting problem, the GOF of training set can reflect the validity of the model.

#### 2.3.4. Initial Values and Dynamo Equations

At 17:42 on 14 February 2020, a man posted on Sina Weibo that his father, an official, had picked him up from Tianmen to Jingzhou using special privileges during the epidemic road closure. Meanwhile, netizens found from the man’s microblog that his total expenses for 2019 were more than 2.86 million yuan. Therefore, many netizens questioned the implementation of local government anti-epidemic measures and the problem of corruption and abuse of power by the man’s father, which led to an outbreak of public opinion. The government department responded and investigated the incident on Feb. 15. Finally, the incident ended with the man posting a letter of apology online and suspension. The initial values involved in this incident are given by Appendix A (Table A1). Based on the cross-validation results and the public sentiment propagation mechanism, dynamo equations are given by Appendix A (Table A1).

## 3. Results

### 3.1. Simulating Results

**Figure 6:**
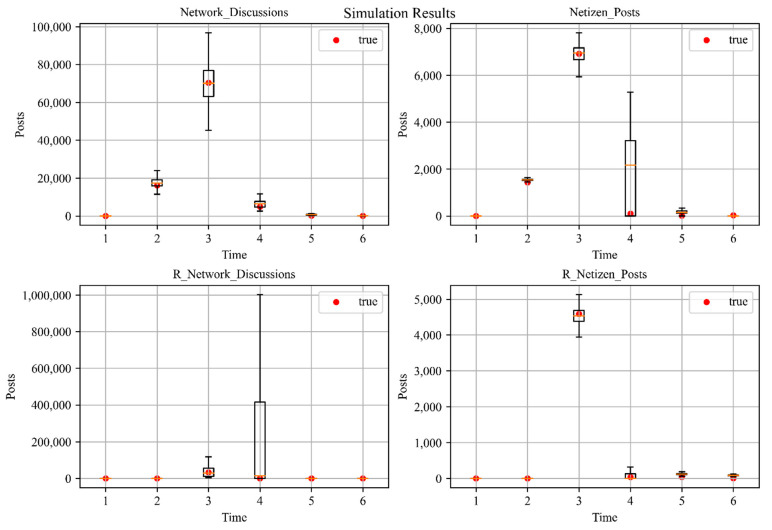
Simulation results, and the box plots show the maximum, minimum, median, and interquartile distance of the data.

We used Python to write the system dynamics simulation code for Table A1; the partial simulation results of the public sentiment are shown in Figure 6. The true values are almost within the box plot, which verifies the validity of the model. In order to guide positive public sentiment, we will analyze the influence of these factors on the evolution process of public sentiment in terms of government behavior, netizen behavior and disaster context.

### 3.2. Decision Analysis

**Figure 7:**
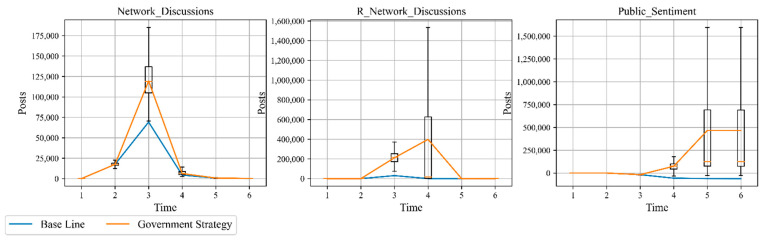
Government analysis.

For better analysis, we used the simulation results of the initial state as a baseline to reveal the influence relationship between variables and system by changing certain variables. The government is not only the guide and stakeholder of public sentiment events, but also the main force in stopping the epidemic. Its behavior has a significant impact on the evolution of public sentiment. In reality, the strength of the government response represented by government postings and media choices is manageable. Therefore, the “Government Strategy” is set: the “R Government Posts” and “R Government Followers” are both adjusted upward by 20% from the baseline to represent the strength of the government response. The simulation results are shown in Figure 7 and Table 4. After the outbreak of public sentiment triggered by COVID-19, the number of overall negative sentiments (Network Discussions) spread significantly as the government response increased. Compared to the “Base Line”, the cumulative number of negative sentiments increased by 52,300, proportional to the strength of the government response. The number of overall positive sentiment (R Network Discussions) increases rapidly as strength of the government response grows. Compared to the “base line”, the number of positive sentiments increased by 579,318, which is proportional to the strength of the government response. It is worth noting that the increase in positive sentiment is much higher than negative sentiment, and the overall number of positive social sentiment (Public Sentiment) increased by 527,018 compared to the “base line”, the overall social sentiment is positive.

**Figure 8:**
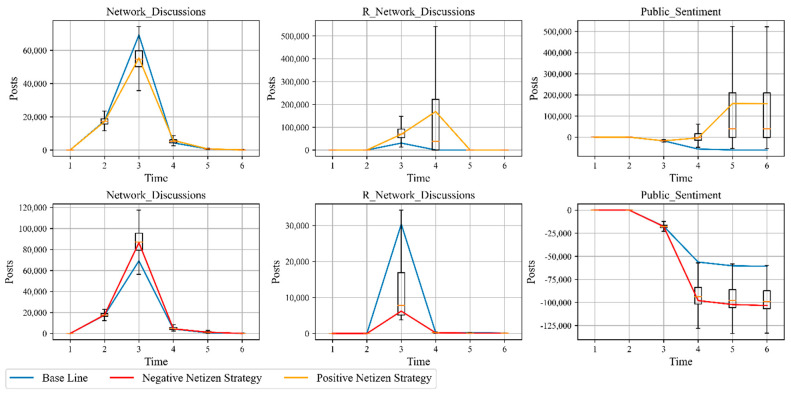
Netizen analysis.

As one of the subjects of the public sentiment system, netizens are both receivers and senders of emotions, and their behavior has an important influence on the evolution of public sentiment. To explore the influence of netizen behavior on the public sentiment system, we set “Positive Netizen Strategy” and “Negative Netizen Strategy”. “Positive Netizen Strategy”: “Netizen Posts” decreased by 20% and “R Netizen Posts” increased by 20% from the baseline to represent the positive individual behavior that suppresses negative sentiment and promotes positive sentiment. “Negative Netizen Strategy”: “Netizen Posts” is increased by 20% and “R Netizen Posts” is decreased by 20% from the baseline to represent the negative individual behavior that promotes negative sentiment and suppresses positive sentiment. The simulation results are shown in Figure 8 and Table 4. In the Positive Netizen Strategy, the curve of negative sentiment (Network Discussions) is significantly lower than the baseline, with a cumulative decrease of 12,585, which is inversely proportional to the positive individual behavior. However, the positive sentiment (R Network Discussions) curve is significantly higher than the baseline, with the cumulative number rising 207,036, proportional to the positive individual behavior. The amount of overall positive social public sentiment (Public Sentiment) increased by 219,561, and the overall social public sentiment is positive. Positive Netizen Strategy shows that positive individual behavior not only inhibits the spread of negative emotions, but also contributes more to the spread of positive emotions, which can increase the level of positive social public emotions in both directions. The simulation results of the “Negative Netizen Strategy” are the exact opposite of the “Positive Netizen Strategy”: negative individual behavior not only increases the spread of negative emotions (18,292 more negative emotions), but also inhibits the spread of positive emotions (24,297 fewer positive emotions), which ultimately leads to a significant increase in overall negative social public sentiment. The opposite simulation results of “Positive Netizen Strategy” and “Negative Netizen Strategy” also verify the robustness of the model.

**Figure 9:**
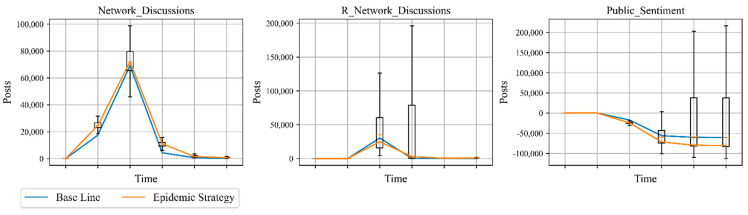
Epidemic analysis.

As the main background of public opinion, disasters are the source of public sentiment events. To explore the impact of disaster context on the public sentiment system, we set the “Epidemic Strategy”: Epidemic Factor increased by 40% to represent a worsening of the epidemic. The simulation results are shown in Figure 9 and Table 4. As the epidemic worsens, the negative sentiment curve is slightly higher than the baseline, proportional to the deterioration of the epidemic. The positive sentiment curve is slightly below the baseline, inversely proportional to the deterioration of the epidemic. Overall social sentiment has declined from the baseline. Although the simulation results all changed compared to the baseline, the magnitude of change was relatively small. This suggests that although disaster environments (epidemics) can have an effect on public sentiment, the effect is relatively mediocre.

## 4. Discussion

According to the results of the model, we have reasons to believe that positive governmental response behavior is beneficial to redeem the positive image of the government among the public and even to reconstruct trust in the negative events derived from the epidemic. Specifically, the government uses e-government media to respond to negative events, and the strength of the government response represented by government postings and media influence can affect the spread of positive public sentiment. The strength of the government response is proportional to the number of positive emotions. Previous findings suggest that proactive actions by authorities can improve negative public sentiment and rumor management in emergency situations and yield positive social utility [33]. Enhancing public relations through social media has proven effective [34]. The simulation results in this paper again validate the above findings. According to Situational Crisis Communication Theory, under intentional crises or accidental crises (e.g., job failure, abuse of authority), rebuild strategies (e.g., aggressive crisis management, satisfactory compensation and punishment, creating an image of positive crisis management) are effective ways to recover or even rebuild reputation [35]. The results of this paper build on the rebuild strategies to further investigate the relationship between positive image promotion behaviors and the spread of positive emotions (reputation or trust). One possible explanation for why increased government responsiveness can facilitate the spread of positive sentiment is that the government, as a network leader, influences the public through high communication activity, credibility, network centrality, and the use of affective, assertive, and linguistic diversity in their online messages [36], and uses mass media to amplify public sentiment [37]. A study showed that exposure to HPV (Human Papilloma Virus) information was associated with the degree of HPV vaccination [38]. This suggests that the government communicates risk to the public through the repetition of information and emphasizes the good attitude of the government in dealing with negative events [39], thus the government has gained the trust of the people. Notably, we also found that government response strength was positively related to negative sentiment, which may seem odd, but similar results have been found in previous studies: higher average positive exposure intensity predicts decreased positive sentiment expression and increased negative sentiment expression [40]. A possible explanation is that the expansion of the scale of the same sentiment discussion might inhibit the expression of the same sentiment and favor a shift to the opposite sentiment [40]. In addition, the expanded scope of government response attracts the expression of negative sentiments from groups that are themselves distrustful of the government. Both explanations are plausible, but the exploration of specific causes and effects requires further research.

In both the Positive Netizen Strategy and the Negative Netizen Strategy, the conclusion is the same: positive individual behavior can inhibit the spread of negative emotions and promote the spread of positive emotions. Specifically, individuals who reduce the transmission of negative emotions and increase the transmission of positive emotions will contribute to an increase in positive social emotions. The results seem obvious, but the implications for the entire public sentiment system (including individuals, government, commercial media, context, etc.) are unknown and meaningful. Previous studies have shown that the formation of identical emotional groups is the result of two factors: emotional contagion and homophily (getting together with people of similar emotions) [41], with the former playing a major role [42]. The conclusion that emotions can be massively contagious on social networks has been extensively verified in previous studies [42,43,44,45], which is an almost confirmed fact, and our simulation results also prove this. Furthermore, previous empirical studies have shown that when individuals reduce their positive emotional expression of events, others’ positive emotional expression decrease and negative expression increase accordingly [45]. This is consistent with the results of our individual behavioral simulations. This suggests that the emotional transmission results of individual behaviors may be related to the initial emotional distribution and the rate of emotional contagion. The final distribution of emotions depends on the distribution of initial emotions [46], and a higher number of individuals unaffected by the emotions of others can effectively reduce aggressive emotions and behaviors [47], and these results provide strong evidence for the influence of initial emotion distribution on emotional contagion. A deeper explanation for this is that strongly connected network structures (e.g., the influence or number of followers of an individual) satisfy the basic requirement for emotional contagion (the possibility of being more widely known) [43]. The contagion rate of emotions depends on the network structure, peer pressure, the nature of the emotion itself, and the characteristics of the individual. Research has shown that contagion of emotions is not only influenced by network structure, but also reinforces it (i.e., people are more willing to express views and empathy with people who have the same emotions) [43]. Peer pressure forces individuals with different emotions to switch to the same emotion. In addition, there are different findings on the contagiousness or influence of emotions in different research contexts. Some studies have suggested that negative emotions are more contagious compared to positive emotions [43,46], but others have taken the opposite view [48], while some have concluded that there is no significant difference in the contagiousness of different emotions [45]. In the Chinese situation, the government is more concerned with building its authority and credibility, so positive sentiment seems to be more popular with the public, as evidenced by the comparison of the effects of the Positive Netizen Strategy and the Negative Netizen Strategy. Finally, the effect of individual characteristics on emotional contagion is very rare in existing studies, but some side evidence suggests that individual personality type [49] (extrovert, introvert) and education [50] have a significant effect on emotional contagion.

Although the disaster context (epidemic) is the source of negative public sentiment events, our simulation results suggest that changes in the disaster context do not seem to have a significant enough impact on the spread of public sentiment. A worse disaster context after a negative event outbreak can slightly promote the spread of negative emotions and slightly inhibit the spread of positive emotions. To date, research on the context of emotional contagion has been relatively sparse, but many studies of sentiment analysis of social networks during COVID-19 seem to be able to detect some patterns. A Twitter analysis of Chinese Netizen sentiment during COVID-19 found that Chinese Netizens’ sentiment was consistently negative, but increased slightly as the outbreak subsided [51]. In addition, many similar studies on Chinese microblogs (WeiBo, Sina, Beijing, China) have found similar results [52,53]. These studies were able to provide evidence for our results. One possible explanation for this result is that negative sentiment events are relatively independent of the disaster context once they are generated. The disaster context provides the initial conditions for the generation of negative emotional events, yet the spread of public sentiment relies heavily on emotional contagion. People are concerned about the problems exposed by the negative events and hope that the government can solve them properly. Therefore, the process of government handling and the process of people’s emotional contagion are the main factors that affect public sentiment. Changes in the disaster context have the potential to influence public sentiment, but changes in the disaster context alone are not enough.

Currently, social media and e-government are playing an increasingly important role in exposing corruption and social problems [54]. It is a challenge for governments and institutions to rebuild their reputation while accepting beneficial improvements from the public. As mentioned at the beginning of this paper, people’s trust in government or institutions plays a very important role in the acceptance of large beneficial public programs (e.g., vaccination). Therefore, we offer some suggestions and reflections based on the results of the study. Social media is a platform for presentation of the image of government, institutions, and local communities [55]. If official communication is marginalized or ignored, it will have serious consequences. As the speed of information interaction increases, governments and institutions first need to move from their former role as broadcasters to information participants and receivers [56]. Secondly, when the government informs the process of handling negative events, it needs to make announcements not only for the public, but also in terms of breadth and depth. Especially in the face of some negative events that may seriously damage the credibility, it is necessary not only to expand the range of users of the announcement as much as possible, but also to give more in-depth interpretations for different government or institutional media (e.g., legal, life, etc.). Finally, governments or institutions need to cultivate media with significant influence and authority over time. The role of these media in guiding sentiment, dispelling rumors, and rebuilding reputations is enormous.

The population is more inclined to follow the emotional expressions of the overall channel than the specific information content in social networks [41], which indicates that people’s emotions can be easily manipulated maliciously. Reducing malicious manipulation of emotions requires both individual and institutional efforts. First of all, the content review mechanism of social media platforms needs to be improved, and comments that are obviously violent, discriminatory and anti-human need to be banned or alerted. Those negative messages that have not been confirmed also need to be informed to each recipient. The purpose of this is to reduce the degree of connectedness of the network structure, making it difficult for emotions to be spread. However, social media platforms do not seem to have an incentive to do so: reducing these posts containing radical statements and emotions would mean a decrease in online social engagement [45]. Therefore, it would be more effective for the entire social platform industry to reach a consensus in this regard. Second, individual characteristics differ in discriminating information and emotions [57], and in general, education is inversely related to online social expression [58]. More educated people care about the content of information when they are exposed to it rather than the subjective emotions of others. This requires governments or institutions to make science knowledge available to the public as much as possible. In addition, the Positive Netizen Strategy seems to be more harmless than the Government Strategy: the Positive Netizen Strategy does not cause an increase in negative emotions, suggesting that improving the quality of individuals and discouraging malicious manipulation of emotions may be a more socially beneficial initiative. Finally, differences in disaster environments may induce different negative public sentiment events. Under an epidemic, negative events expose problems in epidemic preparedness and people focus more on solving existing problems rather than ignoring them. While the mitigation of an epidemic has a significant effect on overall human health, it does not address the specific problems revealed by the negative events. Therefore, additional staffing is needed to specifically address existing problems while ensuring the smooth operation of the epidemic prevention efforts.

## 5. Conclusions

We construct a model of public sentiment transmission under an epidemic based on theories such as system dynamics and cross-validation, and propose a framework that can be used to improve the model. By analyzing the mechanism and influencing factors of online public sentiment dissemination, a specific SD model is constructed to simulate the dissemination process of public sentiment system. Finally, the validity and rationality of the model are proved through real classical cases. On this basis, the in-fluence of governmental behavior, netizens behavior and disaster context on the propagation of public sentiment is analyzed, and a series of conclusions are drawn: (1) increased government response facilitates the spread of positive sentiment; (2) positive individual behavior contributes to an increase in positive sentiment; (3) changes in the disaster environment (epidemic) affect the spread of sentiment, but the effect is mediocre.

This paper provides a new idea for modeling the public sentiment system under sudden disasters, and also provides theoretical support for relevant organizations to take measures to guide public sentiment. However, our model only considers the situation where the government actively deals with negative events. We suggest that future research could be based on this study by including different governmental attitudes in the model and conducting a precise sentiment analysis of the data, which might lead to more interesting and meaningful results.

## Figures and Tables

**Table 1:** Description of data.

Data Name	Pre-Response *n* (%)	Post-Response *n* (%)
Total posts	1,242,287 (57)	935,790 (43)
Total original posts	136,197 (56)	105,969 (44)
Total reposts	1,106,090 (57)	829,821 (43)
Total followers	197 billion (31)	439 billion (69)
Government original posts	3368 (30)	7944 (70)
Government reposts	295,853 (44)	382,303 (56)
Government followers	93 billion (27)	253 billion (73)
Commercial media original posts	15,003 (35)	28,032 (65)
Commercial media reposts	810,237 (64)	447,518 (36)
Commercial media followers	104 billion (36)	186 billion (64)
Netizen original posts	117,826 (63)	69,993 (37)

**Table 2:** Reverse regression outcomes.

Name	Outcomes	*othervar*
Netizen Posts	[4,1,6,6,5,4,2,1,3,5,4.5,4,3,8,3]	RND, RS, EF
Commercial Media Posts	[3,1,7,6,5,4,3,2,4,6,5,4,3,6,2]	RND, RS, EF
Commercial Media Reposts	[4,3,5,3,2.5,2,1.5,1,3,5,4,3,2,9,2]	RND, RS, EF, CMF
Government Reposts	[4,1,7,6,5,4,2,1,3,5,4.5,4,3,6,4]	RND, RS, EF, GF
Mean err	1.85	-
Mean *sharefactor*	[3.75,1.5,6.25,5.25,4.375,3.5,2.125,1.25,3.25,5.25,4.5,3.75,2.75,7.25,2.75]	-
*sharefactor* equation ^1^	NP = a + b * EF + c * SF + d * (RGP + RGR) a = −23328.98463791, b = 3273.79176234, c = 2080.23327003, d = 1002.55401367	-

^1^ The equation uses “Netizen Posts” as the sample data and the abbreviations of the variables in the “Outcomes” column are given in Table A1.

**Table 3:** Cross-validation process and results.

Dependent	Independent ^1^	Train Set R2	Validation Set R2	Equations
NP	[RCMR, CMR, GF, RCMF, RNP, CMP, T, SF, RGR, GR, GFOC]	0.84	0.99	Table A1
CMP	[T, NP, SF, EF, GFOC]	0.99	0.97	
CMR	[T, BMP, RCMF, GP, GR, NP, CMF, SF, RGR, RNP, GF, RCMR, EF, GFOC]	0.86	0.97	
CMF	[T, BMP, GP, RCMF, GR, NP, SF, RCMP, RNP, GF, RCMR, EF, GFOC]	0.91	0.99	
GP	[BMP, T, NP, SF, EF, GFOC]	0.97	0.99	
GR	[BMP, T, RCMF, NP, SF, GF, EF, GFOC]	0.98	0.99	
GF	[BMP, T, RCMF, NP, SF, RCMP, RGR, CMR, RNP, RCMR, GFOC]	0.89	0.99	
RNP	[BMP, T, GP, GR, SF, RGR, RGP, CMR, EF]	0.97	0.99	
RCMP	[GP, SF, RGR, CMR, RGP, GF, EF, GFOC]	0.98	0.99	
RCMR	[T, BMP, RCMF, GP, GR, NP, SF, RGR, CMR]	0.86	0.98	
RCMF	[T, GP, GR, RGF, NP, SF, RGR, CMR, RGP, GF, EF, GFOC]	0.84	0.95	
RGR	[BMP, T, GR, NP, RGF, SF, CMR, GF, EF, GFOC]	0.95	0.99	

^1^ the abbreviations of the variables in the “Independent” column are given in Table A1.

**Table 4:** Analysis results of different strategies.

Strategies	Network Discussions		R Network Discussions		Public Sentiment	
	Simulation	Change	Simulation	Change	Simulation	Change
baseline	91,660	0	30,920	0	−60,771	0
Government Strategy	143,961	+52,300	610,239	+579,318	466,247	+ 527,018
Positive Netizen Strategy	79,075	−12,585	237,957	+207,036	158,789	+219,561
Negative Netizen Strategy	109,953	+18,292	6623	−24,297	−103,345	−42,574
Epidemic Strategy	110,011	+18,351	29,415	−1504	−80,819	−20,047

Note: The simulation data in this table are the cumulative values.

## Data Availability

Data available in a publicly accessible repository that does not issue DOIs. Publicly available datasets were analyzed in this study. This data can be found here: https://s.weibo.com/ and https://ef.zhiweidata.com/library.

## Appendix A

**Algorithm A1.** Reverse Regression
**Input:** Dependent variables data *y*, other independent variables data *othervar*, the number of iterations NI, initial share factor data *sharefactor*, the number of iterations NRI, Maximum and minimum values assumed for *sharefactor* variable *mins* and *maxs*. LD = The number of dependent variables
**for***i***in** 0 to LD: **for** *i2* **in** 0 to NI: **for** *i3* **in** 0 to NRI: *n* = Generate a random number from 0 to the length of *sharefactor* data **for** *i4* **in** *mins* to *maxs*: Convert the *n*th value of *sharefactor* to *i4*. Calculate the goodness-of-fit of the regression of *othervar* and *sharefactor* on the *y* (Equation (5)). Obtain the *sharefactor* with the highest *r2.* Obtain NI *sharefactor* with the highest *r2.* Obtain the LD * NI matrix of *sharefactor* with the highest *r2.* SFMATRIX = the LD * NI matrix of *sharefactor* with the highest *r2* **for** *i1* **in** 0 to LD: **for** *i2* **in** the *i1*th column of SFMATRIX: **for** *i3* **in** numbers from 0 to LD except *i1*: **for** *i4* **in** the *i3*th column of SFMATRIX: Calculate the *r2* of *i2* and *i4* Obtain *i4* with highest r2 Obtain LD—1 *sharefactor* that are most similar to the trend of *i2* in all columns except for column *i1.* Each *sharefactor,* in the *i1*th column of SFMATRIX, gets LD—1 *sharefactor* that are most similar to it Each *sharefactor,* in SFMATRIX, gets LD—1 *sharefactor* that are most similar to it SIMLARM = LD * NI matrix of *sharefactor* with similar trends among different dependent variables Choose the most similar trend in SIMLARM.
**Output:***Sharefactor* with the most similar trend among different dependent variables

**Algorithm A2.** Cross-Validation in the Selection of Linear Models
**Input:** Dependent variables data *y* and independent variables data *id*, deterministic variables DV and uncertain variables UV, the lowest goodness-of-fit (GOF) that can do cross-validation G. IN = None **while** GOF < G: RV = UV except IN DV = add IN to DV **for** *i* **in** RV: Linear regression of the DV and *i* on the dependent variable. Calculate the goodness-of-fit (GOF) Select the *i* with the highest GOF. IN = *i* UV = UV except DV **for** *i* **in** 0 to the number of UV: CV = Combine *i* variables from UV **for** *i2* **in** CV: add *i2* to DV. Separate data by 8:2 as training and validation sets. Train set train model (liner regression). Calculate the validation set error (GOF) using the trained model. Select the model with the highest GOF in the validation set. Select the model with the highest GOF in the validation set.
**Output:** The best model with the highest GOF in the validation set.

**Table A1:** Model equations.

Name	Abbreviation	Equations	Method	Initial Value
Time	T	T = [1,2,3,4,5,6]	-	0
Explanation: Iteration time of the model				
R Discussions	RD	$\frac{dRD}{dT}=RND$	ODE	0
Explanation: Level of network discussion after the government response				
P Discussions	PD	$\frac{dPD}{dT}=ND$	ODE	0
Explanation: Level of network discussion before the government response				
R Government Speed	RGS	Constants: 2	-	-
Explanation: Speed of government response, measured in days				
Epidemic Factor	EF	EF = 0.4 * (NC − 0)/(3887 − 0) + 0.6 * (NEC − 0)/(58,097 − 0)	Min-Max scaling	0
Explanation: Weighted sum of the number of new and existing infections				
Now Confirm	NC	Constants: 5691	-	-
Explanation: Number of new infections				
New Confirm	NEC	Constants: 2644	-	-
Explanation: Number of current infections				
*Sharefactor*	SF	Constants: 3.75	Reverse Regression	-
Explanation: Factors that represent constants during the event, such as the nature of the event itself, the education level of netizens, etc.				
Netizen Posts	NP	NP = POISSON (1874.116 * 1 + 4554.442 * GFOC * T + 0.27 * GF * CMR − 364.268 * GFOC * RGR − 714.778 * T + 86.904 * SF − 0.097 * GF * RCMF + 54.022 * T ** 2 − 4298.99 * GFOC − 11.608 * GFOC * CMR + 1666.766 * GFOC * RCMR − 108.324 * GFOC * RCMF − 1.767 * GFOC * RNP − 5.154 * GR + 0.501 * GR ** 2 − 2.272 * BMP − 17.548 * GFOC * GF − 0.034 * RNP * RCMR)	Liner Regression & poisson	0
Explanation: Number of original postings by netizen				
Commercial Media Posts	CMP	CMP = POISSON (6.022 * 1 + 0.023 * NP * GFOC + 3.065 * SF + 21.104 * GFOC − 29.066 * T + 2.183 * T ** 2 + 15.346 * EF)	Liner Regression & poisson	0
Explanation: Number of posts in commercial media				
Commercial Media Reposts	CMR	CMR = POISSON (78.17 * 1 − 0.034 * RCMF * RCMR − 76.7 * T + 9.187 * SF + 0.324 * CMF * GFOC − 0.177 * CMF + 17.044 * EF − 2.539 * GFOC * GP + 0.001 * GF ** 2 − 0.032 * NP − 0.0 * NP ** 2 + 0.035 * GF * RCMF − 0.002 * GF * RNP − 0.017 * NP * RCMF + 0.004 * CMF * RNP + 0.154 * RNP + 0.032 * NP * T − 0.061 * NP * RCMR + 0.001 * NP * RGR + 0.001 * CMF * NP − 0.011 * CMF ** 2 + 5.016 * T ** 2 + 0.293 * CMF * RCMR + 0.003 * GP * RNP − 0.132 * GP * RCMF + 1.939 * GFOC * RGR − 41.531 * GFOC − 3.366 * GR + 1.197 * BMP − 0.001 * GF * BMP − 0.31 * BMP * T + 17.044 * EF)	Liner Regression & poisson	0
Explanation: Number of commercial media posts retweeted				
Commercial Media Followers	CMF	CMF = 65.935 * 1 − 20.438 * T + 0.416 * SF + 1.441 * T ** 2 + 0.477 * GFOC ** 2 + 0.021 * RCMR * T + 0.0 * GF ** 2 + 0.0 * RCMP * NP + 0.021 * GR * T + 0.024 * RCMP * T − 0.0 * RNP * RCMP − 0.288 * GP * T + 0.097 * GFOC * BMP + 0.023 * NP * T − 0.034 * NP − 0.0 * NP * BMP + 0.037 * GP*RCMR + 0.002 * GFOC * NP − 0.781 * RCMF − 0.037 * GFOC * RCMP + 0.8 * GFOC * T + 0.133 * RCMF * T + 0.002 * RCMF * NP − 0.656 * GFOC * GP + 1.863 * GFOC * RCMR + 0.0 * GP * RNP − 0.029 * GR * RCMR + 0.783 * EF	Liner Regression	0
Explanation: The average number of followers of commercial media involved in the event discussion, indicating the influence of commercial media				
Commercial Media Discussions	CMD	CMD = CMP * CMR	-	0
Explanation: The sum of netizens discussions within commercial media				
Government Posts	GP	GP = POISSON (20.262 * 1 − 1.483 * GFOC ** 2 − 1.384 * SF − 0.0 * BMP ** 2 + 13.428 * GFOC + 0.0 * NP * BMP + 0.215 * GFOC * BMP − 0.344 * BMP − 0.042 * NP * GFOC + 0.254 * BMP * T + −4.084 * EF)	Liner Regression & poisson	0
Explanation: Number of government media postings				
Government Reposts	GR	GR = POISSON (3.462 * 1 + 0.0 * BMP ** 2 − 1.232 * SF + 2.917 * GFOC ** 2 − 0.038 * GF * GFOC + 0.0 * NP ** 2 + 0.001 * GF * RCMF + 0.0 * GF * NP + 0.003 * BMP * RCMF − 0.028 * BMP * T + 0.317 * EF)	Liner Regression & poisson	0
Explanation: Number of government media posts retweeted				
Government Followers	GF	GF = 102.26 * 1 + 0.191 * GFOC * RNP + −9.552 * SF + 0.007 * CMR * BMP + −13.49 * T + 0.64 * T ** 2 + −0.073 * BMP * RGR + 0.001 * RGR ** 2 + −0.02 * GFOC * NP + 0.107 * GFOC * BMP + −0.003 * NP * RCMP + −1.624 * GFOC * RCMF + −0.002 * CMR * RCMF + 0.056 * RCMP * T + 0.011 * RCMP * RGR + −0.028 * RCMR ** 2 + 0.112 * RCMR * T + 1.078 * RCMR + 1.987 * GFOC * RGR + 3.286 * GFOC * RCMR + 0.012 * CMR * RCMR + 0.0 * NP * BMP + −0.45 * RNP + −0.0 * RCMP ** 2 + 0.015 * BMP * RCMP + 0.002 * RNP * RCMF	Liner Regression	0
Explanation: The average number of followers of government media involved in the event discussion, indicating the influence of government media				
Government Discussions	GD	GD = GP * GR	-	0
Explanation: The sum of netizen discussions within government media				
Network Discussions	ND	ND = NP+ BMD + GD	-	0
Explanation: Total postings by netizens, government and commercial media before government response				
R Netizen Posts	RNP	RNP = POISSON (−298.162 * 1 + 4.712 * BMP + 10.359 * SF − 0.007 * RGP * BMP − 0.017 * RGR * CMR + 3.092 * RGP * T + 0.019 * GP * GR + 56.422 * EF)	Liner Regression & poisson	0
Explanation: The number of original posts from netizens after the government response.				
R Commercial Media Posts	RCMP	RCMP = POISSON (−67.288 * 1 + 2.357 * RGP + 1.491 * SF − 0.071 * CMR * GP + 0.432 * GFOC * GF − 0.053 * RGR * RGP + 0.079 * RGR * GP + 0.063 * RGP * CMR + 14.523 * EF)	Liner Regression & poisson	0
Explanation: Number of commercial media postings after government response				
R Commercial Media Reposts	RCMR	RCMR = POISSON (−4.361 * 1 + 0.006 * RCMF * RGR + 1.129 * SF − 0.001 * RGR ** 2 + 0.013 * CMR + 0.02 * GP + 0.007 * RCMF * T − 0.041 * BMP − 0.001 * CMR * BMP + 0.046 * BMP * T − 0.0 * NP * GR)	Liner Regression & poisson	0
Explanation: Retweeted commercial media posts after government response				
R Commercial Media Followers	RCMF	RCMF = 127.299 * 1 − 0.924 * RGF + 2.676 * SF − 43.353 * T − 0.084 * CMR * GR + 0.0 * NP * GR + 0.014 * GP * GR + 0.011 * CMR * GF + 2.827 * T ** 2 + 0.157 * RGF * T − 0.245 * GF * T − 0.025 * RGR * GP + 2.698 * EF + 2.981 * GR * T + 0.003 * RGF * GR + 0.306 * GP * T + 0.29 * GFOC * RGF − 0.06 * RGR * GR − 0.089 * NP − 0.138 * GFOC * RGP + 0.007 * RGR * RGF + 2.698 * EF	Liner Regression	0
Explanation: The average followers of commercial media involved in the discussion of the event after the government response				
R Commercial Media Discussions	RCMD	RCMD = RCMP * RCMR	-	0
Explanation: Total netizen discussion within the commercial media after the government response				
R Government Posts	RGP	RGP = POISSON ([0,305,3,0,0,0])	real data & poisson	0
Explanation: Number of government response postings after the government response				
R Government Reposts	RGR	RGR = POISSON (−23.102 * 1 − 0.0 * NP ** 2 + 3.439 * SF − 0.093 * BMP * T + 0.002 * RGF * CMR − 0.391 * GR * T − 0.0 * RGF ** 2 − 0.001 * GF ** 2 + 0.002 * RGF * GF + 0.002 * CMR * NP + 0.004 * BMP * GR + 0.174 * RGF − 26.594 * GFOC + 0.054 * NP − 0.0 * CMR ** 2 + 2.363 * EF)	Liner Regression & poisson	0
Explanation: Number of government media postings retweeted by netizens after government response				
R Government Followers	RGF	RGF = [0,444,158,0,0,0]	Real Data	0
Explanation: Average number of government media followers after government response				
R Government Discussions	RGD	RGD = RGP * RGR	-	0
Explanation: Total network discussions within government media after government response				
R Network Discussions	RND	RND = RNP+ RBMD + RGD	-	0
Explanation: Total postings and reposts by netizens, government and commercial media after the government response				
Government Focus	GFOC	GFOC = (RGP * RGF − 0)/(253,132 − 0) * 10	Min-Max scaling	0
Explanation: The level of government media involvement in the event discussion.				
Public Sentiment	PS	PS = RD − PD	-	0
Explanation: Propagation of public sentiment before and after the response				

Note: * is for multiplication and ** is for power operations.
